# Effect of input voltage frequency on the distribution of electrical stresses on the cell surface based on single-cell dielectrophoresis analysis

**DOI:** 10.1038/s41598-019-56952-4

**Published:** 2020-01-09

**Authors:** Kia Dastani, Mahdi Moghimi Zand, Hanie Kavand, Reza Javidi, Amin Hadi, Zarrintaj Valadkhani, Philippe Renaud

**Affiliations:** 1 Small Medical Devices, BioMEMS & LoC Lab, School of Mechanical Engineering, College of Engineering, University of Tehran, Tehran, 11155-4563 Iran; 2 School of Mechanical Engineering, Sharif University of Technology, Tehran, Iran; 3 École Polytechnique Fédérale de Lausanne, STI IMT LMIS4, Station 17, CH-1015 Lausanne, Switzerland; 4 Department of Medical Parasitology, Pasteur Institute of Iran, Tehran, Post code: 1316943551 Iran

**Keywords:** Biomedical engineering, Biomedical engineering, Mechanical engineering, Mechanical engineering, Computational science

## Abstract

Electroporation is defined as cell membrane permeabilization under the application of electric fields. The mechanism of hydrophilic pore formation is not yet well understood. When cells are exposed to electric fields, electrical stresses act on their surfaces. These electrical stresses play a crucial role in cell membrane structural changes, which lead to cell permeabilization. These electrical stresses depend on the dielectric properties of the cell, buffer solution, and the applied electric field characteristics. In the current study, the effect of electric field frequency on the electrical stresses distribution on the cell surface and cell deformation is numerically and experimentally investigated. As previous studies were mostly focused on the effect of electric fields on a group of cells, the present study focused on the behavior of a single cell exposed to an electric field. To accomplish this, the effect of cells on electrostatic potential distribution and electric field must be considered. To do this, Fast immersed interface method (IIM) was used to discretize the governing quasi-electrostatic equations. Numerical results confirmed the accuracy of fast IIM in satisfying the internal electrical boundary conditions on the cell surface. Finally, experimental results showed the effect of applied electric field on cell deformation at different frequencies.

## Introduction

One of the applications in which a cell is exposed to an electric field is electroporation, the exact mechanism of which is still uncertain. Controlled transport of molecules across cell membranes is a fundamental part of medical processes: including gene therapy and treatment of diseases such as cancer. Electric field mediated *in vivo* agent delivery protocols, use electroporation (EP) or electropermeabilization to facilitate the transport of molecules into cells. Initially, electroporation was established as a method for gene transport^1–4^, but now it is used for the transport of other molecules such as drugs^5^, ions^6^, dyes^7^, oligonucleotides^8^, proteins^9^, antibodies^10^, RNA^11,12^, etc. Although the mechanism of permeabilization is still not entirely clear, theoretical and experimental evidence shows that the formation of pores in the lipid bilayer membrane is caused by this phenomenon^13^. The electroporation-intermediate delivery system offers various advantages over other competitive methods in being a non-invasive, non-chemical, fast and easily implemented, and a comparatively non-toxic delivery. Electroporation had been used for both *in vitro* and *in vivo* environments. In clinical studies electroporation has been limited to DNA vaccination^14^ and for the treatment of several types of cancer: including skin, lung, breast tumors, bone metastases, and leukemia^15–17^.

Due to the electrodynamic and electrostrictive nature of cells, electromechanical forces are induced on the cell membrane. Cell nuclei show evidence for such electric stress in extreme cases of electrocution lesions, where cell nuclei are stretched in the direction of an applied electric field. In electromechanical force models, defined parameters such as permittivity, conductivity, and material parameters combined with electric field frequency are applied. These modeling tools can be used for accurate prediction of the electric field-induced cell shape deformations^18^.

Several studies have been carried out on electric field-induced cell membrane permeabilization. For example, Neu *et al*.^19^ carried out numerical simulations of radial electromechanical force for toroidal and cylindrical pores in the presence of a uniform electric field and calculated the electrical energy of the nanopores as a function of pore size. Molecular dynamics simulation was performed by Tieleman *et al*.^20^ to show the ability of pore deformations under appropriate electromechanical forces. Some experimental results suggest that the poration of vesicles is highly dependent on the electric field exposure time. Salipante *et al*.^21^ showed that vesicles would rupture in a weak electric field when stressed for a long time, while they survive a strong electric field for short durations. The effect of uniform electric fields on transmembrane potential has been studied numerically by Priva and Gowrisree^22^. Differences between the inner and outer membrane potential have been shown by the comparison of numerical results and analytical values. Needham and Hochmuth^23^ studied the effect of critical external electric field strength on mechanical stretching of the cell membrane. Electroporated cells showed considerable changes in geometrical and electrical properties, which was due to membrane breakdown of lipid vesicles under an external electric field. Numerical and experimental studies on the effect of such cellular modifications were carried out by Oblak *et al*.^24^ and were applied as a basis of an electric field mediated cell separation technique. A novel chip had been designed to calculate the dynamic changes in membrane permeability through electroporation in human cancer cells by He *et al*.^25^ In this paper, dynamics of pore resealing were also investigated and the resealing time constants were calculated for different pulse treatments. In another research, the effect of a cell’s volume fraction, medium conductivity, membrane conductivity, critical transmembrane potential, and cell orientation have been investigated on the effective conductivity of a suspension of electroporated cells^26^. The anisotropic nature of electropermeabilized cells was also evaluated using the finite element method and an analytical approach. Zudans *et al*.^27^ represented a numerical calculation method for estimating the degree of cell electropermeabilization in inhomogeneous electric fields. In the aforementioned studies, the effect of electric field characteristics on cell permeabilization has been studied using different experimental and numerical methods such as molecular dynamics. However, the effect of cell deformation and cell membrane structural changes caused by electrical stresses has not been discussed. Electrical stress distribution over the cell surface and its effect on membrane permeabilization in the presence of an electric field is an imperative process that requires fundamental investigations and should not be overlooked.

The exact mechanism of pore formation across the plasma membrane in the presence of an electric field is not yet well understood. Pipet aspiration is another method used for cell membrane permeabilization. In this method, mechanical tension is applied to cells. Thus it seems that cell deformation caused by mechanical stresses plays an essential role in pore formation on the cell membrane. As mentioned earlier, cells experience such stresses in the presence of an electric field. To better understand the electroporation phenomena, it is necessary to study the effect of electric fields on cells. To this end, the present work studied the behavior of a red blood cell under the application of a non-uniform AC electric field. Electric stresses induced by an electric field acts on the cell surface and cause cell deformation, which may play a role in cell membrane structural changes and its permeability.

Despite the considerable interest in the field of electroporation, to the best of our knowledge, no research group has studied the effect of the applied electric field frequency on the electric stress distribution over a cell’s surface and its subsequent effects on membrane permeabilization analytically. In this paper, the immersed interface method (IIM) was employed to study the electrical stresses generated on a cell membrane exposed to a non-uniform AC electric field. Electrical stresses were calculated on the inner and outer boundaries of the cell membrane using the Maxwell stress tensor (MST). To validate the numerical algorithm, the resultant of electric tension applied on the cell membrane was compared with the dielectrophoretic (DEP) force, which was calculated analytically using effective dipole moment (EDM) approximation. Finally, an experimental analysis was conducted to present the effect of electric stresses on cell deformation at different frequencies.

## Theory

**Figure 1:**
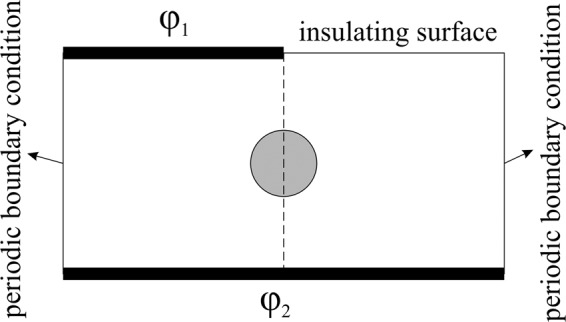
The geometry of enclosure (25 × 50 µm). The applied AC voltage is ${\phi }_{1}({V}_{p-p}=2\,{\rm{V}};\beta =270^\circ )$ and ${\phi }_{1}({V}_{p-p}=2\,{\rm{V}};\beta =0^\circ )$ at the frequency *f*.

We considered a cell with the conductivity of ${\sigma }_{p}$, permittivity of ${\varepsilon }_{p}$, immersed in a dielectric fluid with ohmic conductivity of ${\sigma }_{f}$, and permittivity of ${\varepsilon }_{f}$. A non-uniform AC voltage is applied on the enclosure. The geometry of electrodes is presented in Fig. 1.

The well-known governing quasi-electrostatic equation is seen in Eq. 1, where $\underline{\varepsilon }$ is the complex permittivity that is defined as Eq. 2 with $\omega =2\pi f$ and $f$ being the applied AC voltage frequency.

1 $$\nabla \cdot (\underline{\varepsilon }\nabla \underline{\phi })=0$$

2 $$\underline{\varepsilon }={\varepsilon }_{r}-j{\varepsilon }_{i}=\varepsilon -j\frac{\sigma }{\omega }$$

In the above equation, $\underline{\phi }$ is the complex electric potential that is expressed as Eq. 3.

3 $$\underline{\phi }(t)=\underline{\varphi }(\bar{{\rm{X}}})\,\exp \,(j\omega t)$$

The boundary conditions for the enclosure, shown in Fig. 1, are as follow:

4-a $$\underline{\phi }(\bar{{\rm{X}}})={\underline{\varphi }}_{0}\,\exp \,(j\omega t)\,{\rm{on}}\,{\rm{electrode}}\,{\rm{surface}}$$

4-b $$\frac{\partial \underline{\phi }(\bar{{\rm{X}}})}{\partial \bar{{\rm{X}}}}=0\,{\rm{on}}\,{\rm{insulating}}\,{\rm{surface}}$$

The internal boundary condition (jump condition) on the cell surface is as Eq. 5.

5-a $${\underline{\phi }}_{p}={\underline{\phi }}_{f}$$

5-b $${\underline{\varepsilon }}_{p}\frac{\partial {\underline{\phi }}_{p}}{\partial \bar{n}}={\underline{\varepsilon }}_{f}\frac{\partial {\underline{\phi }}_{f}}{\partial \bar{n}}$$

The effect of electric stress acting on a cell’s surface that is being subjected to an electric field is calculated by using MST. By neglecting the magnetic effects, MST can be calculated for any dielectric particle with ohmic losses using Eq. 6.

6 $$\overleftrightarrow{T}=\varepsilon \bar{E}\otimes \bar{E}-\frac{1}{2}\varepsilon (\bar{E}\cdot \bar{E})I$$

In the above equation, the electric field can be any function of time. According to the literature, when a cell is exposed to an AC electric field, the time scale of its motion is much larger than the electric field frequency. Hence, the time average of MST is calculated, and the time-dependent part is ignored. The electric field can be expressed as Eq. 7.

7 $$\bar{E}(t)={\rm{Re}}[{E}^{M}\,\exp \,(j\omega t)]=\frac{1}{2}(\overline{\underline{E}}(t)+{\overline{\underline{E}}}^{\ast }(t))$$

where ${\bar{E}}^{\ast }$ is the complex conjugate of $\bar{E}$. Maxwell stress tensor for a cell with an ohmic loss can be written as Eq. 8.

8 $$\begin{array}{rcl}\overleftrightarrow{T} & = & \frac{1}{4}\underline{\varepsilon }[(\overline{\underline{E}}\otimes {\overline{\underline{E}}}^{\ast }+{\overline{\underline{E}}}^{\ast }\otimes \overline{\underline{E}})-(\overline{\underline{E}}\cdot {\overline{\underline{E}}}^{\ast })\overleftrightarrow{I}]\\  &  & +\,\frac{1}{4}\underline{\varepsilon }[\overline{\underline{E}}\otimes \overline{\underline{E}}+{\overline{\underline{E}}}^{\ast }\otimes {\overline{\underline{E}}}^{\ast }-\frac{1}{2}(\overline{\underline{E}}\cdot \overline{\underline{E}}+{\overline{\underline{E}}}^{\ast }\cdot {\overline{\underline{E}}}^{\ast })\overleftrightarrow{I}]\end{array}$$

The first part of Eq. 8 is the time average MST, and the second part is its time-dependent part, which goes to zero under the integration over the electric field time period. Thus, the time average of electric stresses acting over the cell surface can be obtained as Eq. 9, in which $T=2\pi /\omega$.

9 $${\bar{f}}_{{\rm{DEP}}}={\overleftrightarrow{T}}_{TA}\cdot \bar{n}=(\frac{1}{T}\,{\int }_{0}^{T}\,\overleftrightarrow{T}\,dt)\cdot \bar{n}=\frac{1}{4}\varepsilon [(\overline{\underline{E}}\otimes {\overline{\underline{E}}}^{\ast }+{\overline{\underline{E}}}^{\ast }\otimes \overline{\underline{E}})-{|\overline{\underline{E}}|}^{2}\overleftrightarrow{I}]\cdot \bar{n}$$

The integration of electric stresses over the cell surface is equal to the net dielectrophoretic (DEP) force exerted on a cell in the presence of the non-uniform electric field. Using the effective dipole moment (EDM) approximation, the time average of DEP force can be calculated using Eq. 10.

10 $$\langle {\bar{F}}_{{\rm{DEP}}}(t)\rangle =2\pi {\varepsilon }_{1}{R}^{3}{\rm{Re}}[\underline{K}(\omega )]\nabla {E}_{{\rm{rms}}}^{2}$$

where $R$ is the cell radius and $\underline{K}(\omega )$ is the Clausius-Mossotti factor, and they depend on the dielectric properties of the cell, suspension media, and the frequency of the applied electric field. Equation 11 defines the Clausius-Mossotti factor for a 2D cell with a complex permittivity of ${\underline{\varepsilon }}_{p}$, immersed in a fluid with the permittivity of ${\underline{\varepsilon }}_{f}$.

11 $$\underline{K}(\omega )=\frac{{\underline{\varepsilon }}_{p}-{\underline{\varepsilon }}_{f}}{{\underline{\varepsilon }}_{p}+2{\underline{\varepsilon }}_{f}}$$

The effective dipole moment is valid only when the cell size is negligible in comparison with the enclosure size or electric field nonuniformity. In such cases, the effect of the cell on the electric potential distribution and the electric field can be neglected. Otherwise, EDM approximation will be inaccurate. The real part of the Clausius-Mossotti factor determines the behavior of the cell in the presence of an electric field.

## Solving Method

To calculate MST on the cell surface, the governing quasi-electrostatic equation in the presence of a cell was first solved. In previous studies, the existence of the cell has been mostly ignored in the computational domain and the cell is replaced by equivalent multipoles. In the present study, fast (augmented) IIM was used to implement internal electric boundary conditions on the cell surface. IIM is a modified finite difference (or finite element) method developed by Z. Li for solving partial differential equations (PDEs) involving interfaces and irregular domains.

In the current study, the governing quasi-electrostatic equation was derived using fast IIM. Fast IIM is a modified immersed interface method for solving PDEs, where the coefficients have a constant value in each subdomain. By considering a 2D elliptic equation involving interface $\Gamma$, $\beta (x,y)$ is assumed to be constant in each domain (Eq. 12).

12-a $$\nabla \cdot (\beta (x,y)\nabla u)=f(x,y)$$

12-b $$\beta (x,y)=\{\begin{array}{ll}{\beta }^{-} & if\,(x,y)\in {\Omega }^{-}\\ {\beta }^{+} & if\,(x,y)\in {\Omega }^{+}\end{array}$$

Internal boundary conditions (jump conditions) on the interface $\Gamma$ are as follow:

13-a $${[\beta \frac{\partial u}{\partial n}]}_{\Gamma }-\nu =0$$

13-b $${[u]}_{\Gamma }=\omega $$

The discrete form of Eq. 12-a can be written as:

14 $$\mathop{\sum }\limits_{k}^{{n}_{s}}\,{\gamma }_{k}{U}_{i+{i}_{k},j+{j}_{k}}={f}_{ij}+{C}_{ij}$$

In the above equation, ${C}_{ij}$ is the correction term. If the coefficient $\beta$ is constant in Eq. 12, or is constant in each of the domains, or there is only the singular force on the interface, then the finite difference coefficients of Eq. 14 are simplified to the standard 5-point finite difference scheme. However, a correction term still needs to be added, which is due to a jump in the value of coefficient $\beta$ or a source distribution along the interface. Thus, the discrete form of Eq. 12 can be written as Eq. 15, where Δ_*h*_ is the discrete Laplacian operator and ${C}_{ij}$ is the correction term.

15 $${\Delta }_{h}{U}_{ij}=\frac{{f}_{ij}}{{\beta }_{ij}}+{C}_{ij}$$

Based on the standard 5-point finite difference stencil centered at $(i,j)$, if all five grid points are on the same side of the interface, point $(i,j)$ is called a regular grid point. Otherwise, it is an irregular grid point. The correction term at regular points is zero; grid points that are away from interfaces. At irregular points, the correction term is calculated in terms of the first and the second surface derivatives of $\nu$ and $\omega$ at the control point $(X,Y)$ on the interface using Eq. 16, where $\chi$ is the interface curvature.

16 $$\begin{array}{rcl}{C}_{ij} & = & {a}_{2}\omega +{a}_{12}\frac{{\nu }^{-}}{\beta }+({a}_{6}+{a}_{12}\chi ^{\prime\prime} )\omega ^{\prime} \\  &  & +\,{a}_{10}\omega ^{\prime\prime} +\frac{1}{\beta }({a}_{4}+({a}_{8}-{a}_{10})\chi ^{\prime\prime} )\nu \\  &  & +\,{a}_{8}(\frac{[f]}{\beta }+\frac{\sigma \omega }{\beta }-\omega ^{\prime\prime} )\end{array}$$

Coefficients $${a}_{n}$$ are:

17 $$\begin{array}{ll}{a}_{1}=\sum _{k\in {K}^{-}}\,{\gamma }_{k} & {a}_{2}=\sum _{k\in {K}^{+}}\,{\gamma }_{k}\\ {a}_{3}=\sum _{k\in {K}^{-}}\,{\xi }_{k}{\gamma }_{k} & {a}_{4}=\sum _{k\in {K}^{+}}\,{\xi }_{k}{\gamma }_{k}\\ {a}_{5}=\sum _{k\in {K}^{-}}\,{\eta }_{k}{\gamma }_{k} & {a}_{6}=\sum _{k\in {K}^{+}}\,{\eta }_{k}{\gamma }_{k}\\ {a}_{7}=\sum _{k\in {K}^{-}}\,\frac{1}{2}{\xi }_{k}^{2}{\gamma }_{k} & {a}_{8}=\sum _{k\in {K}^{+}}\,\frac{1}{2}{\xi }_{k}^{2}{\gamma }_{k}\\ {a}_{9}=\sum _{k\in {K}^{-}}\,\frac{1}{2}{\eta }_{k}^{2}{\gamma }_{k} & {a}_{10}=\sum _{k\in {K}^{+}}\,\frac{1}{2}{\eta }_{k}^{2}{\gamma }_{k}\\ {a}_{11}=\sum _{k\in {K}^{-}}\,{\xi }_{k}{\eta }_{k}{\gamma }_{k} & {a}_{12}=\sum _{k\in {K}^{+}}\,{\xi }_{k}{\eta }_{k}{\gamma }_{k}\end{array}$$

In the above equations, the values of ${\gamma }_{k}$ are determined according to the applied finite difference stencil and ${K}^{\pm }=\{k:({\xi }_{k},{\eta }_{k})\,{\rm{is}}\,{\rm{on}}\,{\rm{the}}\,\pm \,{\rm{side}}\,{\rm{of}}\,\Gamma \}$. ${\xi }_{k}$ and ${\eta }_{k}$ are local coordinates of the grid points’ finite-difference stencil centered at $(i,j)$ in the system that its origin is the control point $(X,Y)$ on the interface in the normal and tangential directions.

The augmented variable can be considered as ${[{u}_{n}]}_{\Gamma }=g$. The discrete values of $\omega$ and $g$ on the interface at each of the control points are $W={[{W}_{1},{W}_{2},\cdots ,{W}_{{n}_{b}}]}^{T}$ and $G={[{G}_{1},{G}_{2},\cdots ,{G}_{{n}_{b}}]}^{T}$, respectively and ${n}_{b}$ is the number of control points. Since the correction term is a linear combination of $\{{G}_{k}\}$ and $\{{W}_{k}\}$, the matrix form of Eq. 15 can be written as follow:

18 $$AU+BG=F+{B}_{1}W={F}_{1}$$

The discrete form of jump condition (Eq. 13-a) is obtained as Eq. 19, where ${U}_{n}$ is the discrete value of $\partial u/\partial n$ and $V$ is the discrete values of $\nu$ on the interface.

19 $$[\beta {U}_{n}]-V={\beta }^{+}{U}_{n}^{+}-{\beta }^{-}{U}_{n}^{-}-V$$

Equation 19 must be satisfied along the interface $\Gamma$. ${U}_{n}$ was calculated at the control point $(X,Y)$, along the inner side of the interface using a weighted least square interpolation as Eq. 20.

20 $$\frac{\partial {U}^{-}}{\partial n}(X)=\mathop{\sum }\limits_{k=0}^{{k}_{s}-1}\,{\gamma }_{k}{U}_{{i}^{\ast }+{i}_{k},{j}^{\ast }+{j}_{k}}-C$$

In Eq. 20, *C* is the correction term. By solving the following system of equations, the coefficients $\{{\gamma }_{k}\}$can be determined. In the case of an undetermined system of equation, singular value decomposition (SVD) must be applied.

21 $$\begin{array}{ll}{a}_{1}+{a}_{2}=0 & {a}_{3}+{a}_{4}=1\\ {a}_{5}+{a}_{6}=0 & {a}_{7}+{a}_{8}=0\\ {a}_{9}+{a}_{10}=0 & {a}_{11}+{a}_{12}=0\end{array}$$

By using a weighted least square interpolation, the discrete form of jump condition was obtained as Eq. 22.

22 $$\begin{array}{c}({\beta }^{+}-{\beta }^{-})\,\mathop{\sum }\limits_{k=0}^{{k}_{s}-1}\,{\gamma }_{k}{U}_{{i}^{\ast }+{i}_{k},{j}^{\ast }+{j}_{k}}\\ \,+\,({\beta }^{+}-({\beta }^{+}-{\beta }^{-})({a}_{4}+{a}_{8}\chi ^{\prime\prime} -{a}_{10}\chi ^{\prime\prime} ))g\\ \,-\,({\beta }^{+}-{\beta }^{-}){a}_{12}g^{\prime} \\ \,-\,\nu -({\beta }^{+}-{\beta }^{-})\bar{C}\cong 0\end{array}$$

where $\bar{C}$ is expressed as:

23 $$\bar{C}={a}_{2}\omega +{a}_{6}\omega ^{\prime} -{a}_{8}(\omega ^{\prime\prime} -[\frac{f}{\beta }])+{a}_{10}\omega ^{\prime\prime} +{a}_{12}\omega ^{\prime} \chi ^{\prime\prime} $$

Finally, the jump condition was obtained as Eq. 24. By solving Eqs. 18 and 24, the distribution of *u* can be calculated.

24 $$EU+TG=PV+QW$$

**Figure 2:**
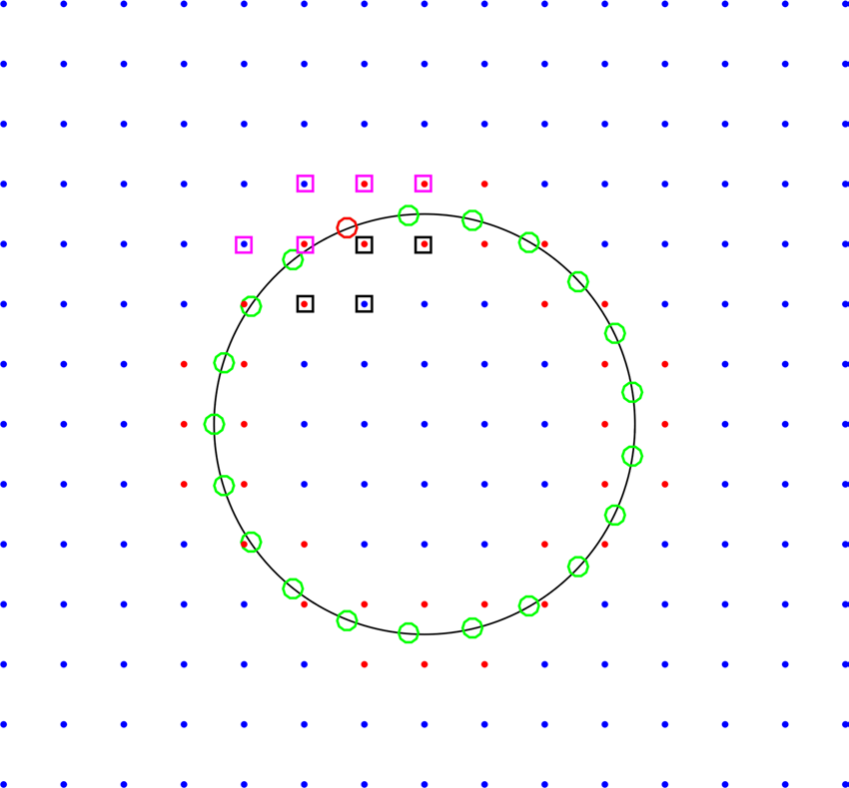
The mesh considered for the numerical solution. Regular, irregular, and control points are shown by blue, red, and green points, respectively. The red circle is the control point at which $\partial u/\partial n$ is interpolated. Squares show the points involved in interpolation.

To examine the accuracy of fast IIM, Eq. 12 was solved using IIM for ${\beta }^{-}=1$, ${\beta }^{+}=1000$, and ${f}^{-}={f}^{+}=2000$ and the results were compared to the exact solution. The boundary conditions and the jump conditions were determined using the exact solution. Figure 2 shows the considered uniform mesh and also the regular and irregular grid points.

**Figure 3:**
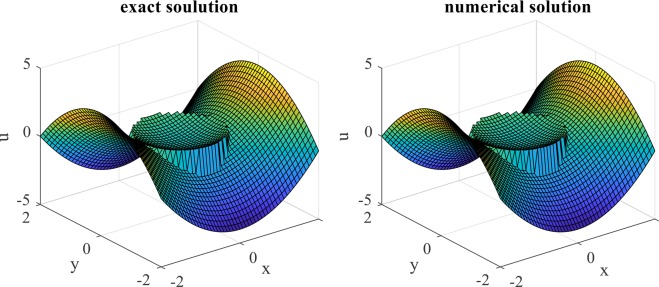
Plots of the exact and numerical solutions.

**Figure 4:**
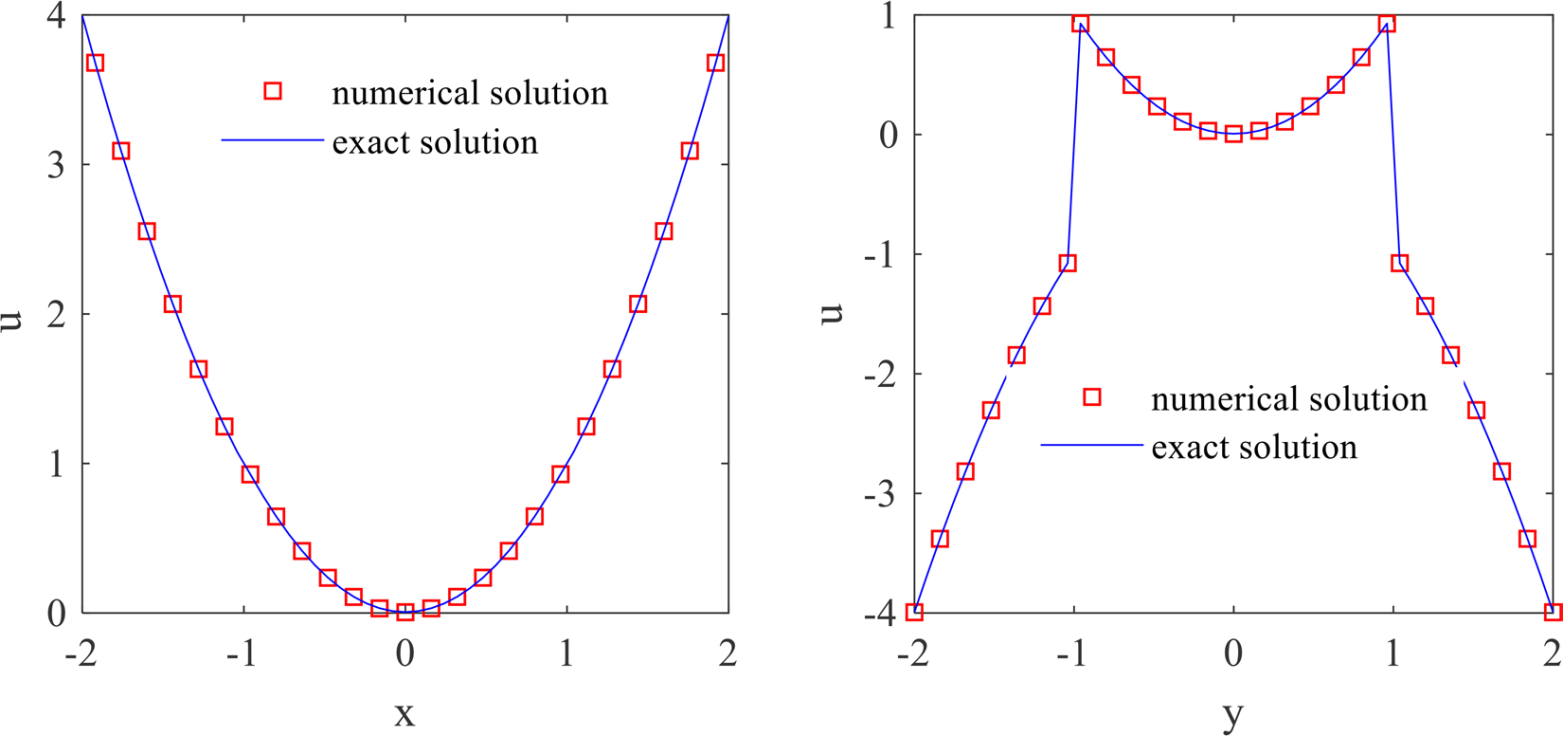
Comparing the numerical and exact solutions at (a) $x=0$ and (b) $y=0$.

The numerical and exact solutions of the problem are plotted in Fig. 3. In Fig. 4, the numerical and the exact solutions are compared at lines $x=0$ and $y=0$. These figures show that the solution algorithm is accurate, and the internal boundary conditions are well satisfied. To calculate the electric stresses acting over the cell surface in the presence of an AC electric field, the electric field discontinuity on the cell membrane must be calculated accurately. These results show that IIM is an appropriate numerical method, which helps to handle the field parameter discontinuity in the solution domain using a uniform mesh.

By separating the real and the imaginary parts of Eq. 1, the following equation is obtained:

25 $$[\nabla \cdot ({\varepsilon }_{r}\nabla {\phi }_{r})+\nabla \cdot ({\varepsilon }_{i}\nabla {\phi }_{i})]+j[\nabla \cdot ({\varepsilon }_{r}\nabla {\phi }_{i})-\nabla \cdot ({\varepsilon }_{i}\nabla {\phi }_{r})]=0$$

If the dielectric properties of the cell are isotropic, it can be concluded that:

26-a $${\nabla }^{2}{\varphi }_{r}=0$$

26-b $${\nabla }^{2}{\varphi }_{i}=0$$

Also, the jump condition on the cell surface can be presented as follow:

27-a $$({\varepsilon }_{r,f}\frac{\partial {\varphi }_{r,f}}{\partial \bar{n}}-{\varepsilon }_{r,p}\frac{\partial {\varphi }_{r,p}}{\partial \bar{n}})+({\varepsilon }_{i,f}\frac{\partial {\varphi }_{i,f}}{\partial \bar{n}}-{\varepsilon }_{i,p}\frac{\partial {\varphi }_{i,p}}{\partial \bar{n}})=0$$

27-b $$({\varepsilon }_{r,f}\frac{\partial {\varphi }_{i,f}}{\partial \bar{n}}-{\varepsilon }_{r,p}\frac{\partial {\varphi }_{i,p}}{\partial \bar{n}})-({\varepsilon }_{i,f}\frac{\partial {\varphi }_{r,f}}{\partial \bar{n}}-{\varepsilon }_{i,p}\frac{\partial {\varphi }_{r,p}}{\partial \bar{n}})=0$$

By considering the augmented variables, ${g}_{1}$ and ${g}_{2}$, as follow:

28-a $$\frac{\partial {\phi }_{r,p}}{\partial \bar{n}}-\frac{\partial {\phi }_{r,f}}{\partial \bar{n}}={g}_{1}(\eta )$$

28-b $$\frac{\partial {\phi }_{i,p}}{\partial \bar{n}}-\frac{\partial {\phi }_{i,f}}{\partial \bar{n}}={g}_{2}(\eta )$$

The discrete form of Eq. 26 can be obtained as Eq. 29, where ${G}_{1}$ and ${G}_{2}$ are discrete values of ${g}_{1}$ and ${g}_{2}$.

29-a $$A{\varphi }_{r}+B{G}_{1}={F}_{1}$$

29-b $$A{\varphi }_{i}+B{G}_{2}={F}_{2}$$

The discrete jump condition of Eq. 27 was derived as Eq. 30, where ${F}_{3}$ and ${F}_{4}$ are zero in this problem.

30-a $${H}_{1}{\varphi }_{r}+{D}_{1}{G}_{1}+{H}_{2}{\varphi }_{i}+{D}_{2}{G}_{2}={F}_{3}$$

30-b $${H}_{3}{\varphi }_{r}+{D}_{3}{G}_{1}+{H}_{4}{\varphi }_{i}+{D}_{4}{G}_{2}={F}_{4}$$

Finally, Eq. 31 can be solved to obtain the electric potential distribution in the enclosure in the presence of a cell.

31 $$\left[\begin{array}{cccc}A & [0] & B & [0]\\{[0]} & A & [0] & B\\ {H}_{1} & {H}_{2} & {D}_{1} & {D}_{2}\\ {H}_{3} & {H}_{4} & {D}_{3} & {D}_{4}\end{array}\right]\left[\begin{array}{c}{\varphi }_{r}\\ {\varphi }_{i}\\ {G}_{1}\\ {G}_{2}\end{array}\right]=\left[\begin{array}{c}{F}_{1}\\ {F}_{2}\\ {[0]}\\ {[0]}\end{array}\right]$$

## Experimental Method

### Microfluidic device fabrication

The dielectrophoretic force generating chip used for cell shape deformation analysis was comprised of parallel planar electrodes with a 25 μm gap, located on glass substrates. Electrodes were patterned on 550 μm thick float glass using photolithography (Microchemicals AZ1512 positive photoresist on MicroChem LOR5A lift-off resist), metal deposition (20/200 nm Ti/Pt sputtered in Alliance-Concept DP 650), and lift-off (Microposit 1165 remover). Channels were then patterned on top of the electrodes using 20 μm layer of SU-8 (MicroChem SU8 2000 permanent epoxy resist). Chips were reversibly sealed by a flat piece of PDMS (polydimethylsiloxane, Dow Corning Silgard 184). The use of 2D electrodes on transparent substrates makes the observations of cell deformation possible under the predefined parameters.

### Electronics

The electrodes were driven by signals in the range of 50 kHz to 20 MHz generated by a function generator (Aim-TTi TG2000 20 MHz DDS) and amplified with a custom electronic board to a final voltage of 3.5 Vp-p in the channel.

### Suspension media

Healthy human red blood cell sample was obtained from a medical laboratory with the donor’s written informed consent and immediately processed. The use of donor blood samples for scientific research was approved by the research ethics committee, Pasteur Institute of Iran, reference number IR.PII.REC.1397.016. The blood samples (from one person obtained on different dates, initial hematocrit level = 41.2%) were centrifuged to remove plasma and white blood cells, washed three times in the suspension buffer, re-suspended in the suspension buffer to a final concentration of 1 × 10^6^ cell/mL, and immediately applied for DEP analyses. Suspension buffer was prepared by diluting PBS (Phosphate Buffered Saline, Gibco) in deionized water and adjusted to a conductivity of 280 μS/cm and a pH of 7.01 (measured by a Mettler Toledo pH/conductivity meter). Osmolarity was adjusted to the physiological range (280 mOsm) by sucrose. To reduce cell sticking to the channel, 0.1% w/w BSA (Bovine Serum Albumin, Sigma-Aldrich) was used. RBC suspension was injected into the microfluidic device and some time was given to the cells to settle before DEP analysis, which was carried out at ambient temperature. In each set of experiments, 8 cells were analyzed per each frequency.

## Results and Discussion

### Numerical solution

**Figure 5:**
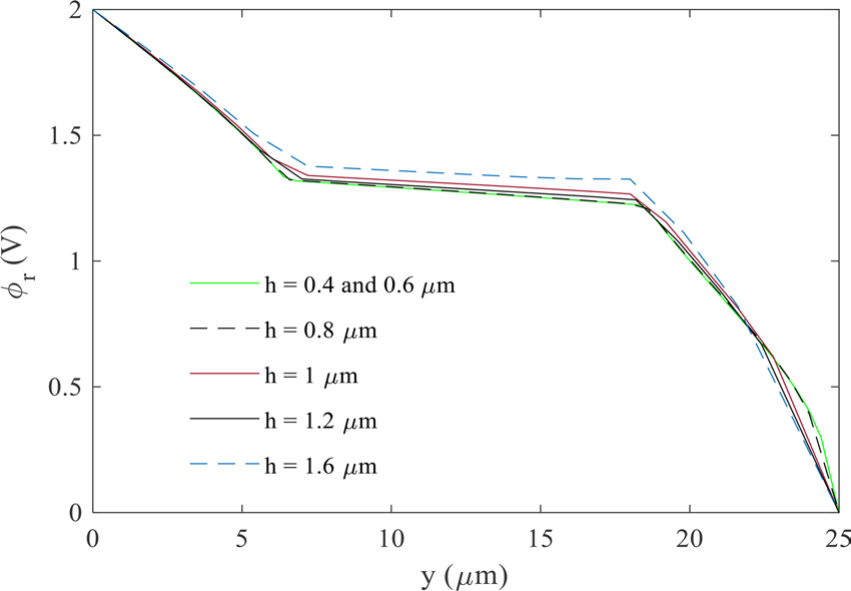
The real part of electric potential (V) at $x=25$ μm and ${\varepsilon }_{p}/{\varepsilon }_{f}=0.01$.

Demonstrating convergence in numerical analysis is the first step in solving problems. The mesh convergence study, for different mesh sizes, is shown in Fig. 5. This figure displays the electrostatic potential at $x=25$ μm (middle vertical line in Fig. 1). In addition to the mesh size, the numbers of interpolation and control nodes are also important for the convergence of IIM. The number of control nodes was determined according to the mesh size. Sixteen nodes were used for interpolation. According to Fig. 5, the mesh size $h=0.6$ μm was shown appropriate for a good convergence. As illustrated in these figures, the electric potential on the boundary of the cell is continuous while the electric field, which is the electric potential gradient, is discontinuous.

**Figure 6:**
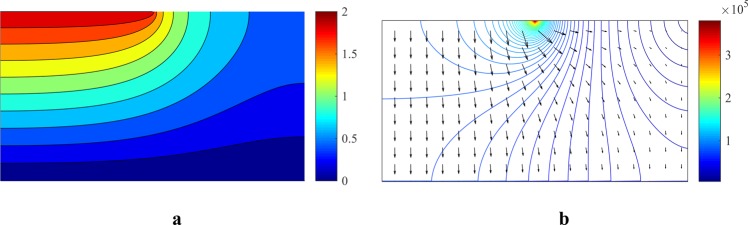
(**a**) Electrostatic potential distribution (the real part) and (**b**). Electric field distribution (the real part) in the absence of a cell in the channel.

Figure 6 presents the electrostatic potential distribution and the electric field in the channel without the presence of a cell.

**Figure 7:**
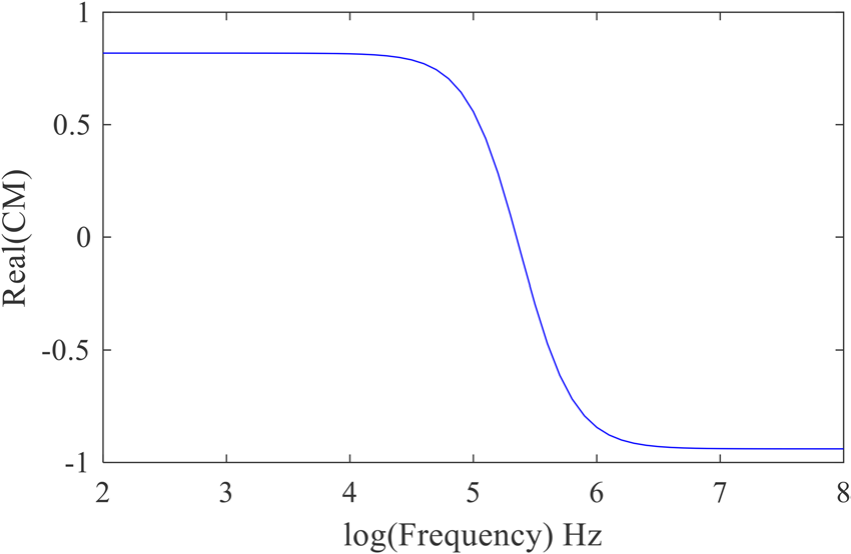
The effect of frequency on the real part of the Clausius-Mossotti coefficient for ${\sigma }_{p}={10}^{-3}{\rm{S}}/{\rm{m}}$, ${\varepsilon }_{p}=2.5{\varepsilon }_{0}$, ${\sigma }_{f}={10}^{-4}{\rm{S}}/{\rm{m}}$, and ${\varepsilon }_{f}=80{\varepsilon }_{0}$.

The value and the direction of the DEP force depend on the value and the sign of the real part of the Clausius-Mossotti coefficient. The dielectrophoretic spectrum of a cell, which shows the effect of the frequency on the real part of the coefficient, provides information about the cell’s electrical structure. In addition to the frequency, the real part of the Clausius-Mossotti coefficient depends on the dielectric properties of the cell and the suspension buffer. In Fig. 7, the effect of frequency on the real part of the Clausius-Mossotti is shown.

**Figure 8:**
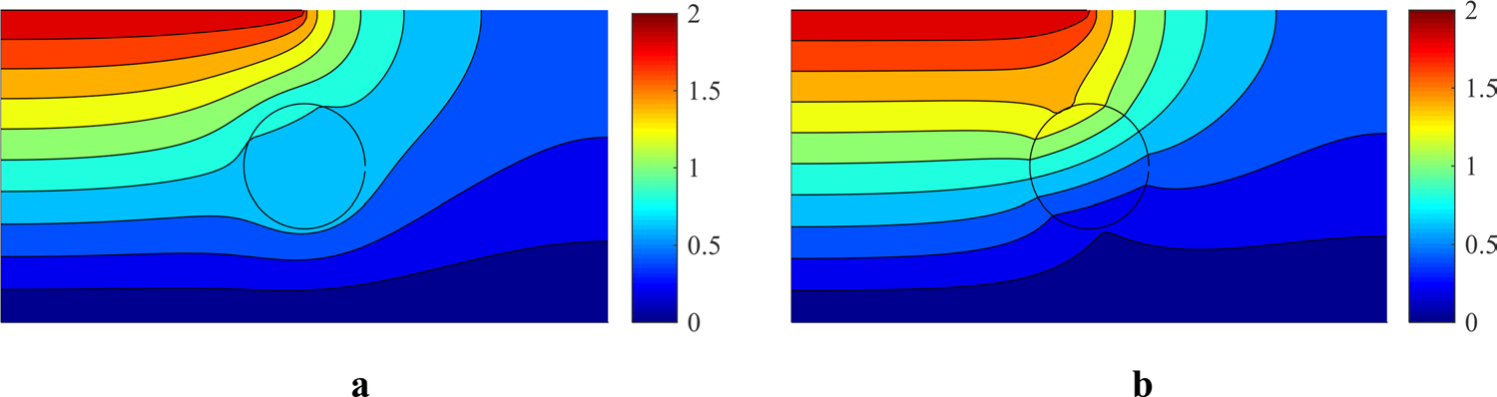
The effect of a cell on the electrostatic potential distribution (the real part) at the frequency of (**a**) 10 KHz and (**b**) 10 MHz for ${\sigma }_{p}={10}^{-3}{\rm{S}}/{\rm{m}}$, ${\varepsilon }_{p}=2.5{\varepsilon }_{0}$, ${\sigma }_{f}={10}^{-4}{\rm{S}}/{\rm{m}}$ and ${\varepsilon }_{f}=80{\varepsilon }_{0}$.

The results from the solved quasi-electrostatic equations, with the presence of the cell in the channel, are shown in Fig. 8. In this case, a cell is considered with an electric conductivity of ${\sigma }_{p}={10}^{-3}\,{\rm{S}}/{\rm{m}}$ and an electric permeability of ${\varepsilon }_{p}=2.5{\varepsilon }_{0}$, floating in a dielectric medium with an electric conductivity of ${\sigma }_{f}={10}^{-4}\,{\rm{S}}/{\rm{m}}$, and an electric permeability of ${\varepsilon }_{f}=80{\varepsilon }_{0}$.

**Figure 9:**
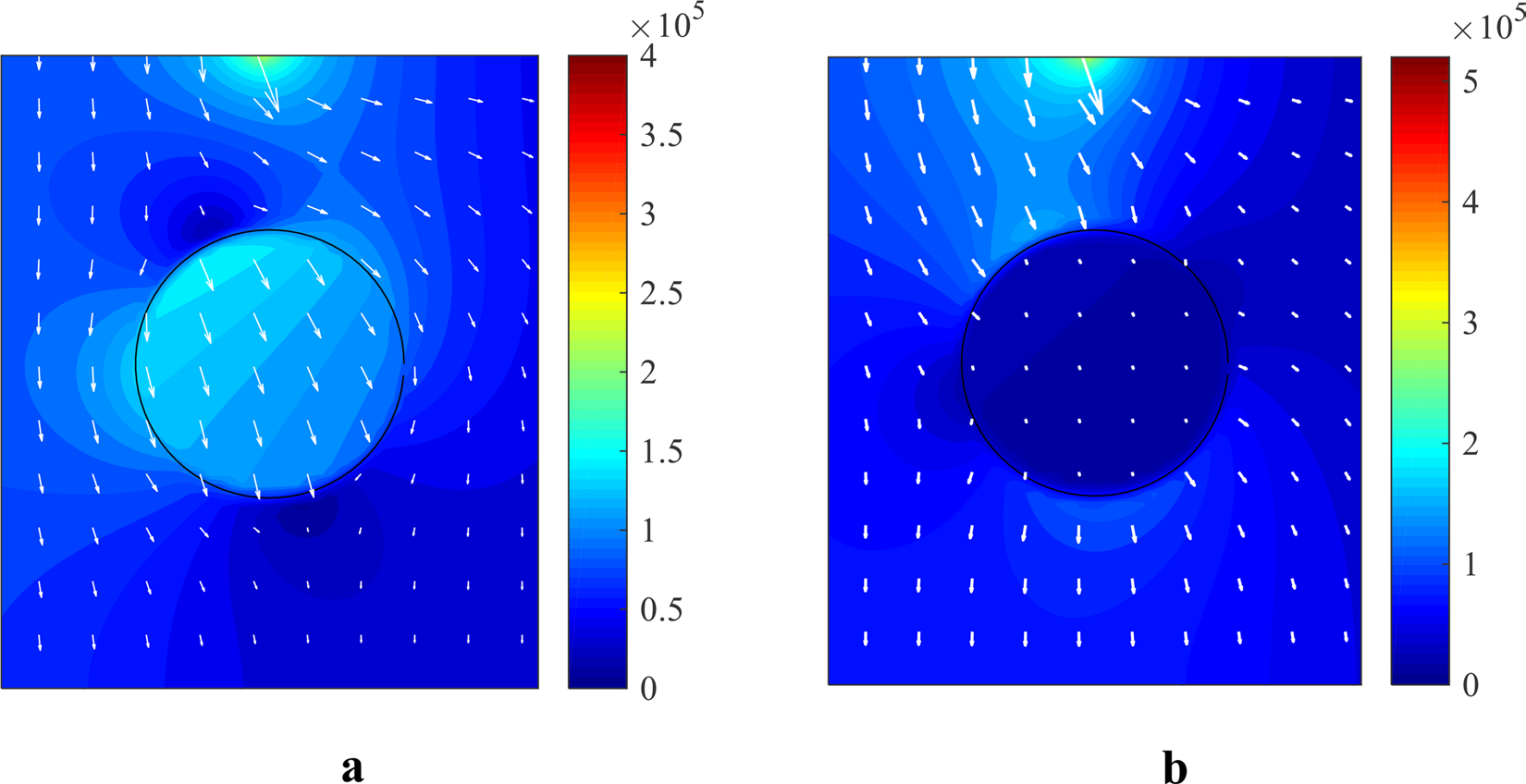
The electric field (the real part) around a cell with ${\sigma }_{p}={10}^{-3}\,{\rm{S}}/{\rm{m}}$ and ${\varepsilon }_{p}=2.5{\varepsilon }_{0}$, immersed in a medium with ${\sigma }_{f}={10}^{-4}\,{\rm{S}}/{\rm{m}}$ and ${\varepsilon }_{f}=80{\varepsilon }_{0}$ at the frequency of (**a**) 10 KHz and (**b**) 10 MHz.

As can be seen in Fig. 7, the behavior of the cell changes in the frequency range from 1 kHz to 10 MHz. Thus, to determine the effect of frequency on cell behavior, the equations were solved for this range of frequencies. The electric potential and the electric field distribution in the presence of the cell at the frequencies of 10 kHz and 10 MHz were calculated. Different effects of a cell on the distribution of the electric potential and the electric field at the two discussed frequencies are presented in Figs. 8 and 9.

**Figure 10:**
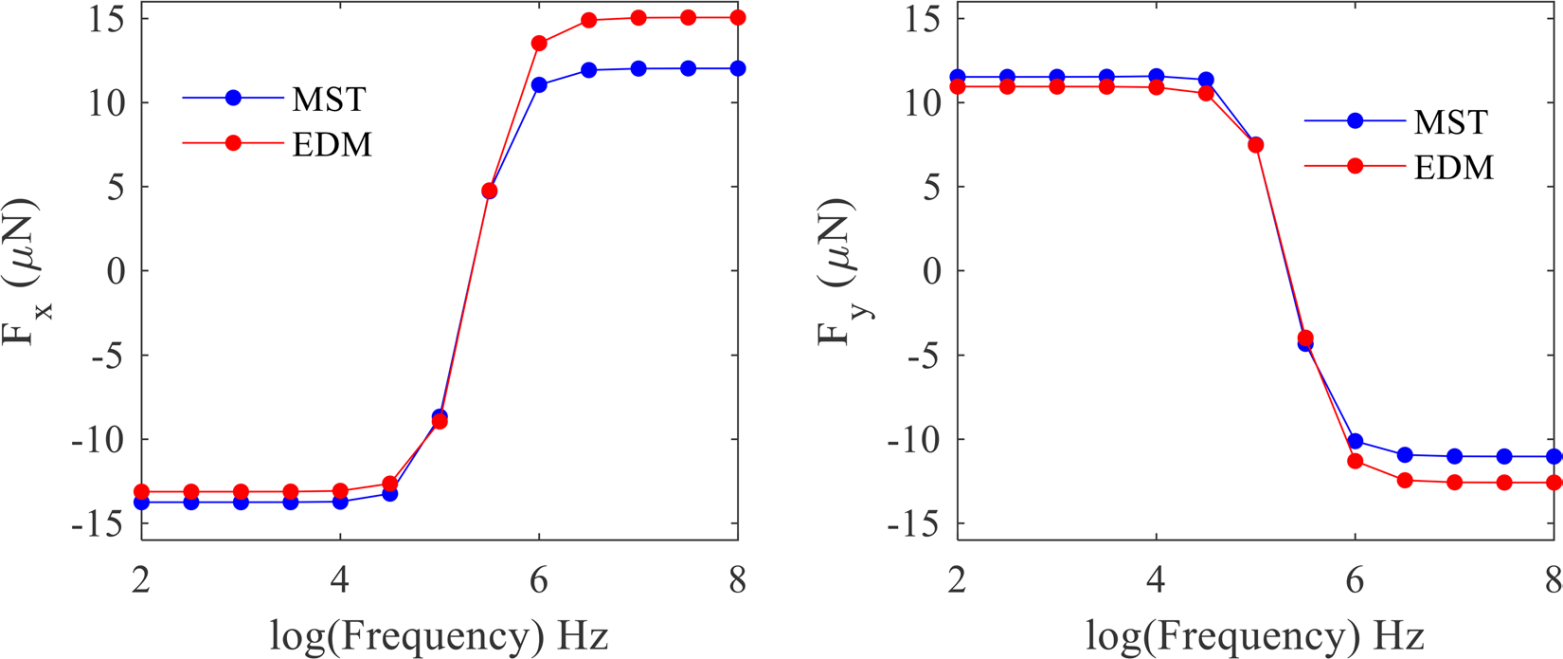
The DEP resultant force exerted on the cell at various frequencies for ${\sigma }_{p}={10}^{-3}{\rm{S}}/{\rm{m}}$, ${\varepsilon }_{p}=2.5\,{\varepsilon }_{0}$, ${\sigma }_{f}={10}^{-4}{\rm{S}}/{\rm{m}}$ and ${\varepsilon }_{f}=80{\varepsilon }_{0}$.

To validate the numerical solution, the DEP force was calculated and compared using both the Effective Dipole Moment (EDM) method and also by integrating the MST on the cell surface (radius = 5 µm) at different frequencies (Fig. 10). As shown, the results indicate the validation of the numerical solution and the inaccuracy of the EDM method. The AC electric field bears the advantage of a controlled DEP force due to its ability in tuning the applied frequency (Fig. 10).

**Figure 11:**
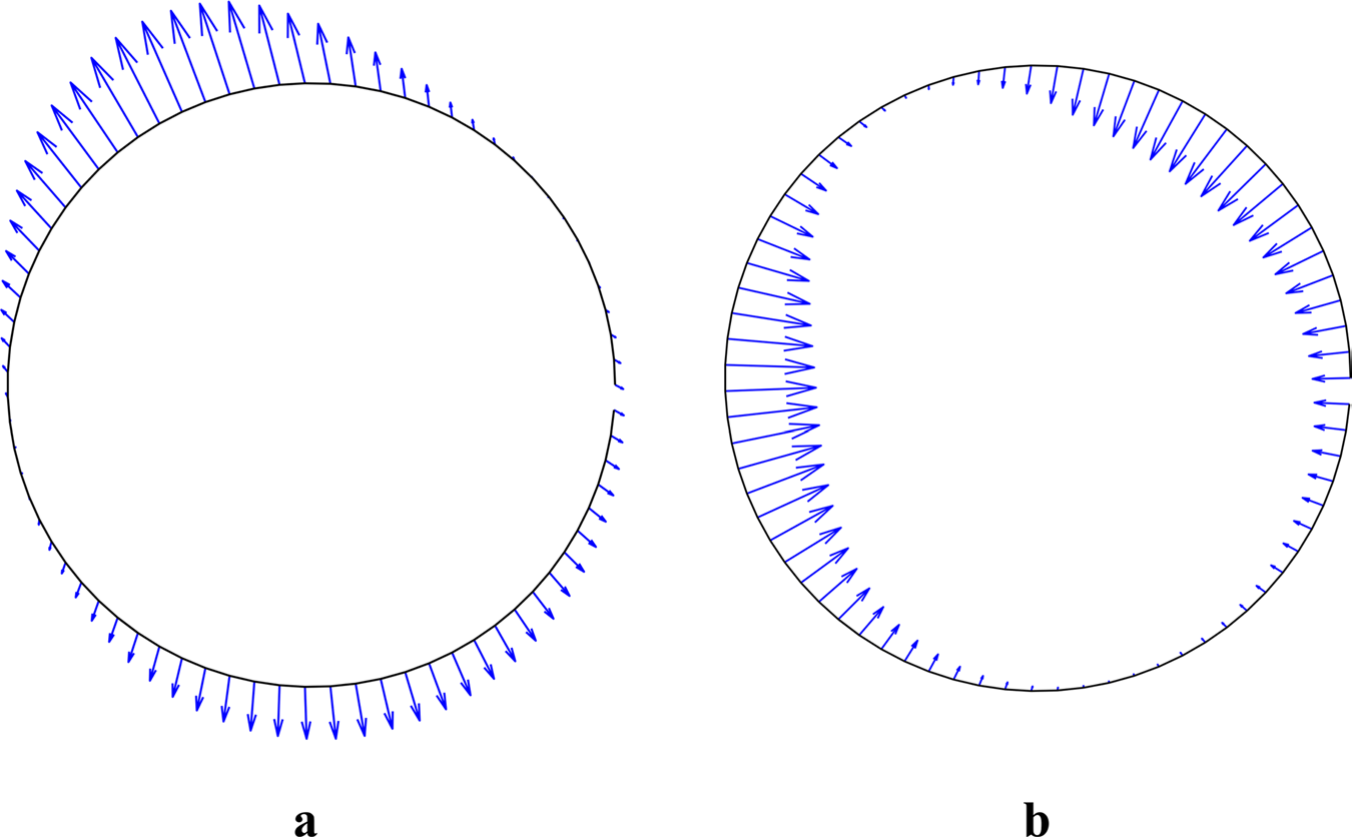
Time average electric stresses acting over the cell surface with ${\sigma }_{p}={10}^{-3}{\rm{S}}/{\rm{m}}$ and ${\varepsilon }_{p}=2.5{\varepsilon }_{0}$ immersed in a medium with ${\sigma }_{f}={10}^{-4}{\rm{S}}/{\rm{m}}$ and ${\varepsilon }_{f}=80{\varepsilon }_{0}$ at the frequency of (**a**) 10 KHz and (**b**) 10 MHz.

The time average of electric stresses on the cell’s surface is shown at the frequencies of 10 kHz and 10 MHz (Fig. 11). It can be concluded that the effect of a non-uniform AC electric field on the cell is different at various frequencies. Therefore, it is expected that the time average of the DEP force should also be different at these two frequencies.

**Figure 12:**
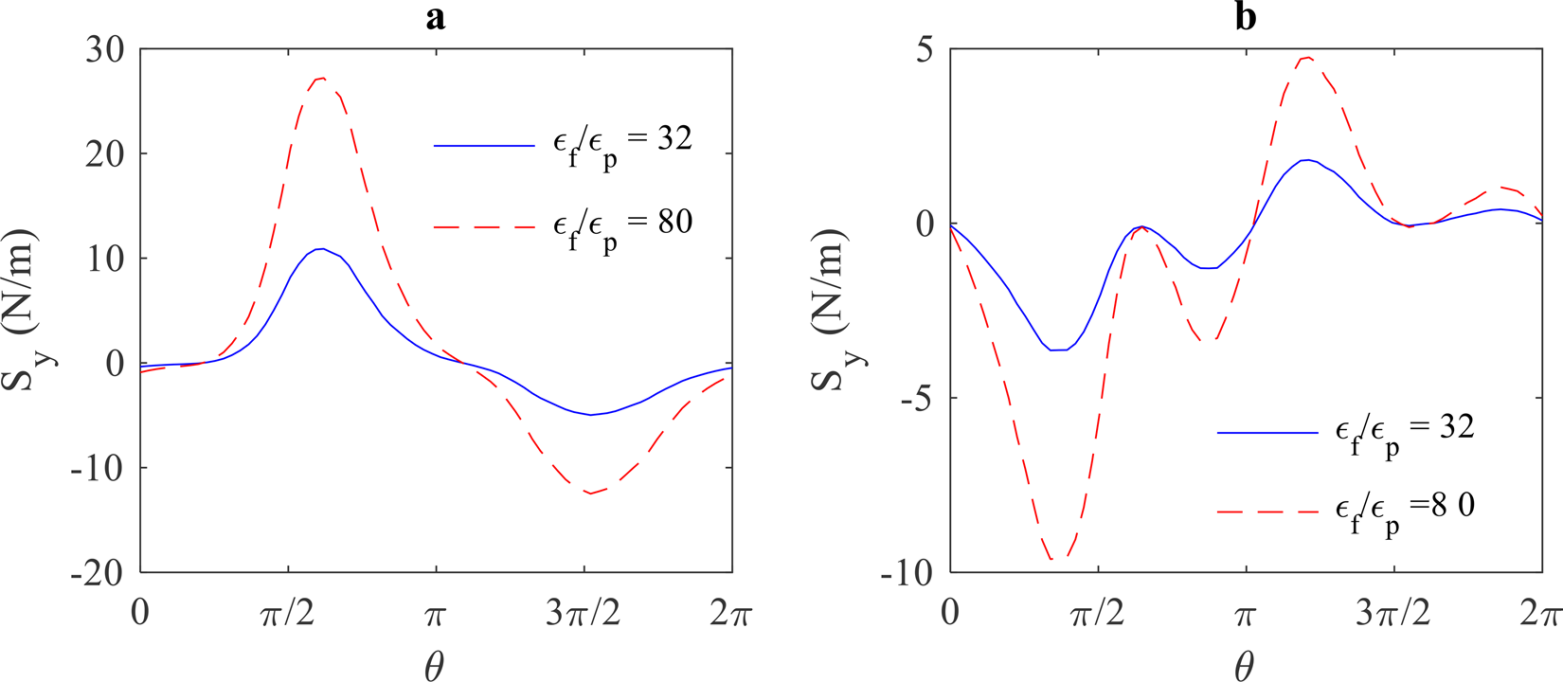
The effect of buffer solution’s permittivity on the magnitude of electric stresses (y-component) at the frequency of (**a**) 10 kHz and (**b**) 10 MHz.

**Figure 13:**
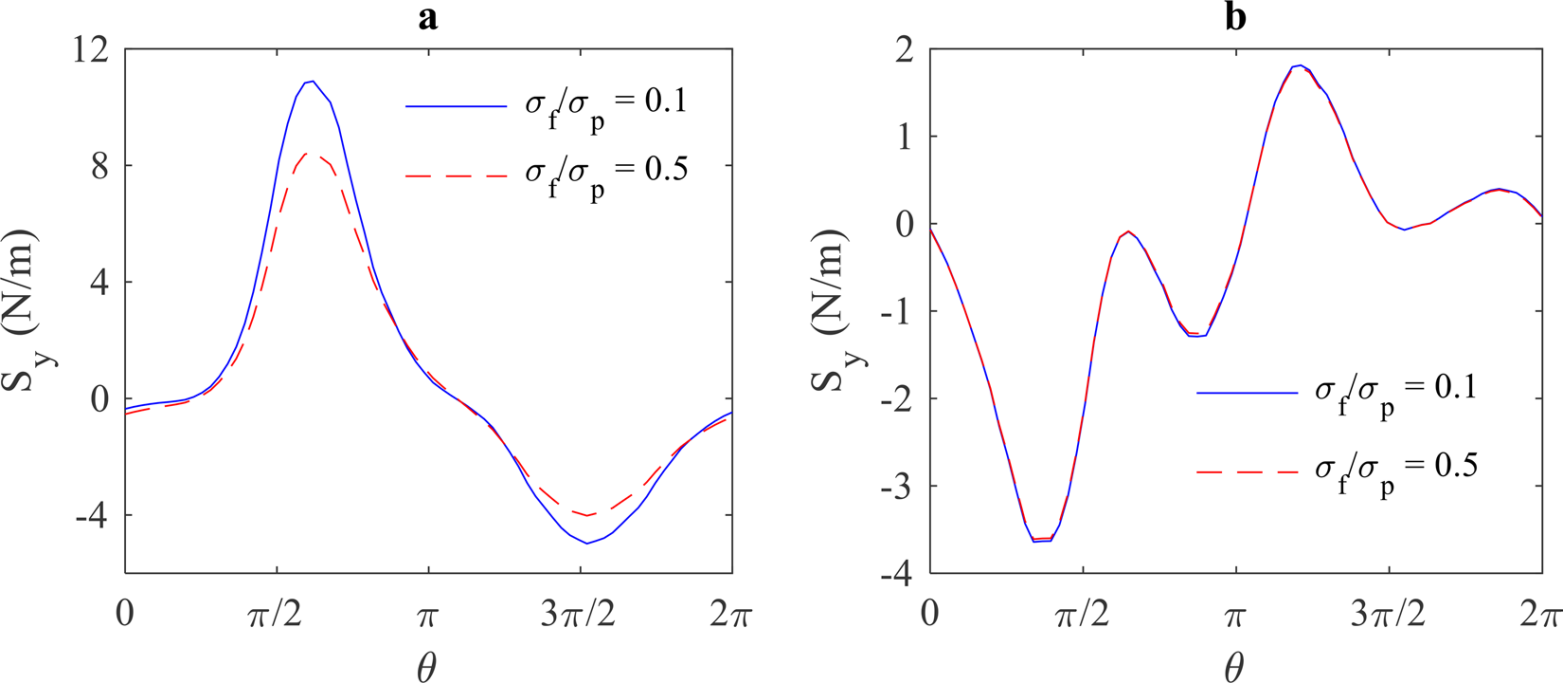
The effect of buffer solution’s conductivity on the magnitude of electric stresses (y-component) at the frequency of (**a**) 10 kHz and (**b**) 10 MHz.

The effects of buffer solution’s dielectric properties on the magnitude of electric stresses at the frequencies 10 kHz and 10 MHz is shown in Figs. 12 and 13, respectively. From these figures, it can be seen that the magnitude of electric stresses increases as the ratio of ${\varepsilon }_{f}/{\varepsilon }_{p}$ increases, and it decreases as the ratio of ${\sigma }_{f}/{\sigma }_{p}$ increases. It can also be seen that the conductivity of the buffer solution has no effect on electric stresses at high frequencies.

**Figure 14:**
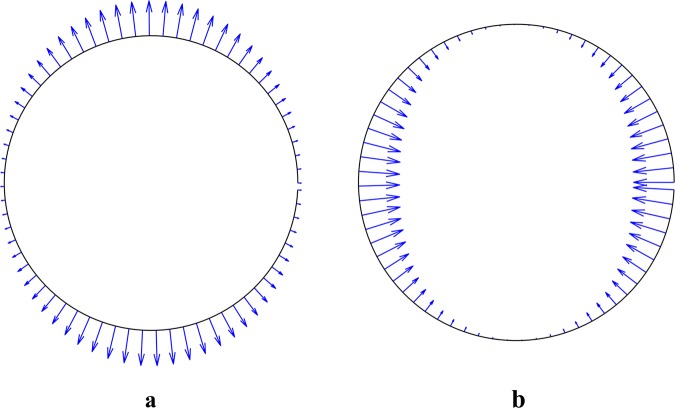
Time average electric stress acting over the cell surface exposed to a uniform AC electric field (**a**) tensile stress at low-frequency (10 kHz) and (**b**) compressive stress at high-frequency (10 MHz).

The electric stresses from uniform electric field acting on the cell surface are shown in Fig. 14. In this figure, the electric field is considered to be in the y-direction. As can be seen, the resultant force exerted on a cell in a uniform electric field is zero, which is because the stress distribution over the cell surface is symmetrical.

### Experimental results

**Figure 15:**
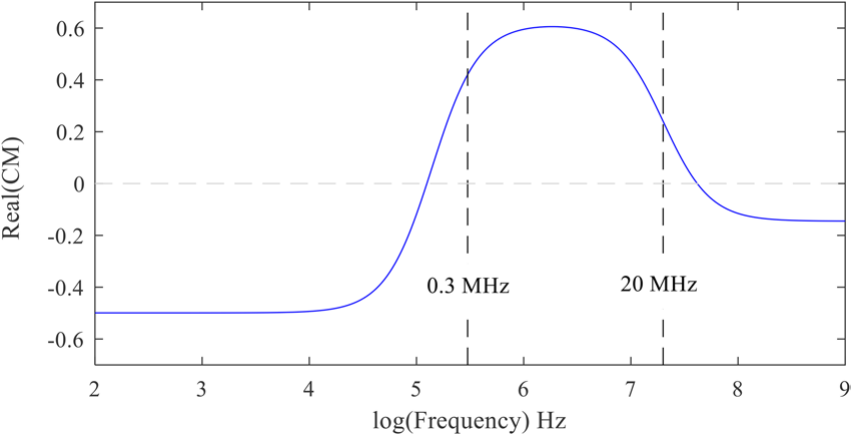
The real part of the Clausius-Mossotti factor for red blood cells versus frequency.

Figure 15 shows the real part of the Clausius-Mossotti coefficient versus the applied electric field frequency to a red blood cell using the single-shell model with the parameters found in the literature^28,29^. The diagram was attained utilizing the spherical single-shell model defined by Gimsa *et al*.^30^.

In the previous section, the distribution of the electrical stresses on the cell surface was calculated. As shown (Fig. 14), these stresses are tensile and compressive at low and high frequencies, respectively. In this section, cell deformation in the presence of the electric field at various frequencies was investigated experimentally.

**Figure 16:**
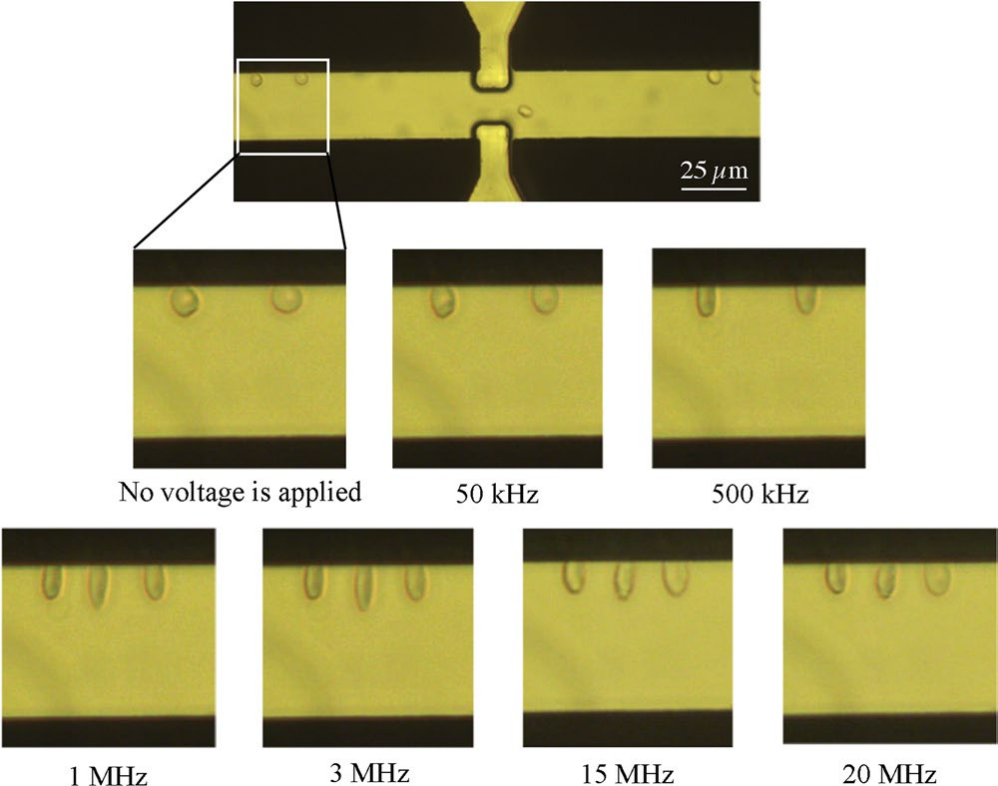
Red blood cell deformation is caused by the electric stresses acting over cell surface at different frequencies.

Figure 16 shows cell deformation under the application of an AC electric field at different frequencies from 5 kHz to 20 MHz. According to Fig. 15, RBCs are in nDEP regime at the frequency of 50 kHz, while they are in pDEP regime at the frequencies of 500 kHz, 1 MHz, 3 MHz, 15 MHz and 20 MHz. As shown in Figs. 11 and 14, when a cell is affected in nDEP regime, the electrical stresses act on the cell surface in the form of tensile forces, whereas in pDEP regime, there will be compressive stresses exerted over the cell surface. According to Fig. 16, cell elongation increases as the frequency increases from 500 kHz to 1 MHz. The maximum cell deformation occurred at the frequency of 1 MHz, which corresponds to the peak of Clausius-Mossotti coefficient versus frequency plot (Fig. 15), while cell deformation was reduced at the frequencies of 15 MHz and 20 MHz. Also seen in Fig. 16, cells deformed under the effect of tensile and compressive electric stress distribution at the frequency of 50 kHz and 500 kHz, respectively. The cell deformation under tensile and compressive electrical stresses looks almost alike (in terms of elongation along y-axis). Looking at the tensile and compressive electrical stresses (Figs. 11 and 14), this result was expected. Based on these experimental results, no comprehensive discussion can be provided about the distinctive effects of tensile and compressive electrical distributions on the cell surface. Despite the electrical stresses magnitude, their tensile or compressive distribution on the cell surface may have an important effect on membrane structural changes. Further research is needed to address this issue.

**Figure 17:**
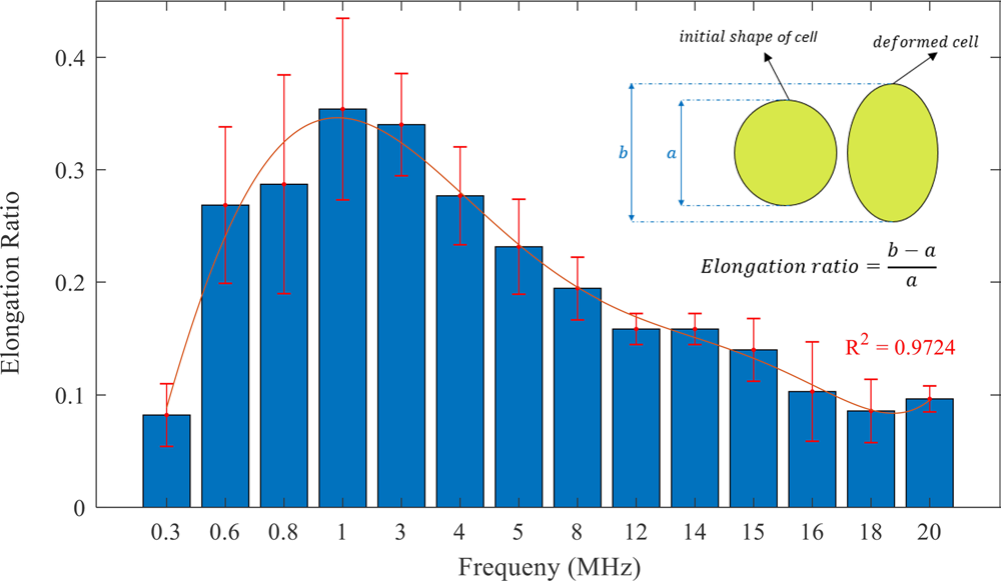
Red blood cell elongation ratio was calculated based on cell deformation in the direction of the electric field lines and plotted at different frequencies. A polynomial of degree 6 is fitted to the experimental results and the error bars represent the standard deviation.

Based on the above results, it can be concluded that the applied electric field frequency has a significant effect on cell deformation. To better show this effect, statistical analysis was conducted at different frequencies (from 300 kHz to 20 MHz) and the elongation ratio (i.e. the ratio of the cell elongation to its initial size) was calculated. The results from this analysis (Fig. 17), show that by increasing the applied electric field frequency up to 1 MHz, cell elongation ratio increases and reaches its maximum value. By further increasing the frequency, cell elongation ratio decreases. These observations match well with Clausius-Mossotti curve versus frequency plot (Fig. 15); by increasing the frequency from 300 kHz to 20 MHz the CM coefficient increases up to 1 MHz and reaches its maximum value and then decreases.

## Conclusion

Cells are exposed to electromagnetic fields in various applications such as electroporation, electrofusion, etc. To better study these phenomena, it is necessary to understand the interaction between cells and electromagnetic fields. One of the most prominent electrokinetic effects of electric fields on cells is dielectrophoresis; a phenomenon that has found many biomedical applications. Previous works, mostly studied the dielectrophoretic effect of electric fields on a group of cells. Therefore, the present study focused on analyzing the behavior of single cells in an electric field. When a cell is exposed to an electric field, local changes in the electric potential distribution are induced. As a result, electrical stresses are exerted on the cell surface. These electrical stresses, in turn, lead to cell deformation and its membrane structural changes that trigger cell membrane permeabilization. Electrical stresses depend on the dielectric properties of cells, buffer solution, and the applied electric field characteristic. In the present study, the effect of applied voltage frequency on the distribution and the magnitude of electrical stresses exerted on a cell surface was numerically and experimentally studied. To study the interaction of cells and electric fields numerically, a novel numerical approach in solving the quasi-electrostatic equations in the presence of cells using the immersed interface method was applied. It was shown that the applied voltage frequency has a significant effect on cell deformation. Furthermore, it was shown that the effect of applied voltage frequency on cell deformation is consistent with the Clausius-Mossotti coefficient curve. In conclusion, a new approach to study the electroporation phenomenon was introduced. Developing an appropriate mechanical model for cell membranes can facilitate the studies conducted on the effects of electric field-induced cell deformations and membrane structural changes, which help to make biomedical processes and applications efficient. Moreover, the electrokinetic interaction of molecules and cells needs to be analyzed to study the transport of molecules across the cell membrane. The numerical approach applied in this research can help in analytical studies on cells and molecules interactions.

### Ethics statement

In this research, healthy human red blood cells were used. Blood samples have been used in this research in an ethical manner. Also, authors confirm that all experiments were performed in accordance with relevant guidelines and regulations. Healthy human blood sample was obtained from a medical laboratory with the donor’s written informed consent. The experimental procedure was approved by the research ethics committee, Pasteur Institute of Iran, reference number IR.PII.REC.1397.016.
